# Clustering the autisms using glutamate synapse protein interaction networks from cortical and hippocampal tissue of seven mouse models

**DOI:** 10.1186/s13229-018-0229-1

**Published:** 2018-09-15

**Authors:** Emily A. Brown, Jonathan D. Lautz, Tessa R. Davis, Edward P. Gniffke, Alison A. W. VanSchoiack, Steven C. Neier, Noah Tashbook, Chiara Nicolini, Margaret Fahnestock, Adam G. Schrum, Stephen E. P. Smith

**Affiliations:** 1 Center for Integrative Brain Research, Seattle Children’s Research Institute, Seattle, WA USA; 2 Department of Immunology, Mayo Clinic College of Medicine, Rochester, MN USA; 3 Present address: Department of Biomedical Engineering, UT Austin, Austin, TX USA; 4 Present address: Nanostring, Seattle, WA USA; 5 Present address: Department of Cancer Immunology and Virology, Dana-Farber Cancer Institute, Boston, MA USA; 6 Present address: Department of Medicine, Harvard Medical School, Boston, MA USA; 7 Present address: Broad Institute of Harvard and MIT, Cambridge, MA USA; 8 Department of Psychiatry & Behavioural Neurosciences, McMaster University, Hamilton, ON Canada; 9 Departments of Molecular Microbiology & Immunology, Surgery and Bioengineering, University of Missouri, Columbia, MO USA; 10 Department of Pediatrics and Graduate Program in Neuroscience, University of Washington, Seattle, WA USA

## Abstract

**Background:**

Autism spectrum disorders (ASDs) are a heterogeneous group of behaviorally defined disorders and are associated with hundreds of rare genetic mutations and several environmental risk factors. Mouse models of specific risk factors have been successful in identifying molecular mechanisms associated with a given factor. However, comparisons among different models to elucidate underlying common pathways or to define clusters of biologically relevant disease subtypes have been complicated by different methodological approaches or different brain regions examined by the labs that developed each model. Here, we use a novel proteomic technique, quantitative multiplex co-immunoprecipitation or QMI, to make a series of identical measurements of a synaptic protein interaction network in seven different animal models. We aim to identify molecular disruptions that are common to multiple models.

**Methods:**

QMI was performed on 92 hippocampal and cortical samples taken from seven mouse models of ASD: Shank3B, Shank3Δex4-9, Ube3a^2xTG^, TSC2, FMR1, and CNTNAP2 mutants, as well as E12.5 VPA (maternal valproic acid injection on day 12.5 post-conception). The QMI panel targeted a network of 16 interacting, ASD-linked, synaptic proteins, probing 240 potential co-associations. A custom non-parametric statistical test was used to call significant differences between ASD models and littermate controls, and Hierarchical Clustering by Principal Components was used to cluster the models using mean log_2_ fold change values.

**Results:**

Each model displayed a unique set of disrupted interactions, but some interactions were disrupted in multiple models. These tended to be interactions that are known to change with synaptic activity. Clustering revealed potential relationships among models and suggested deficits in AKT signaling in Ube3a^2xTG^ mice, which were confirmed by phospho-western blots.

**Conclusions:**

These data highlight the great heterogeneity among models, but suggest that high-dimensional measures of a synaptic protein network may allow differentiation of subtypes of ASD with shared molecular pathology.

**Electronic supplementary material:**

The online version of this article (10.1186/s13229-018-0229-1) contains supplementary material, which is available to authorized users.

## Background

As the incidence of autism spectrum disorder (ASD) has climbed over the past decades to 1 in 59 children [1], next-generation sequencing studies have described likely causative mutations in hundreds of genes, each accounting for < 0.1–1% of the total autistic population [2–4]. Additional factors such as maternal immune activation [5], maternal anti-brain antibodies [6], chemical exposures [7], and polygenetic inheritance of a susceptible genetic background [8] all likely contribute to the development of ASD on an individual-by-individual basis. Thus, much like cancer, ASD is an individually rare, collectively common disorder with a shared diagnostic phenotype: reduced interest in social interaction, reduced communication, and increased stereotyped or repetitive interests and behaviors [9].

The fact that ASD is a diagnostic entity, with a common set of behavioral impairments shared among patients, has led to the widespread hypothesis that other disease mechanisms must also be shared among patients at the level of anatomy [10], neural circuits [11], genetic networks [12, 13], or molecular pathways [14]. Along these lines, a few clear themes have emerged from combining diverse lines of evidence: the immune system is likely involved, with immune-mediated risk factors (reviewed in [15]), and abnormal peripheral [16] and central ([17, 18], but see [19]) inflammatory phenotypes present. Gene regulatory pathways are clearly implicated by genetic studies, as a large percentage of ASD-linked genes are transcription factors, chromatin remodelers, or translational regulators [4, 12]. Synaptic proteins have also been implicated by genetic studies, and by the fact that one unifying feature of animal models of ASD has been disrupted synaptic transmission [20] (although note that the specific nature of the disruption varies greatly between models, or even between brain regions in the same model, discussed below). Recently, unifying theories of ASD have proposed that disruptions to activity-dependent, homeostatic neuronal processes are an underlying characteristic of ASDs [21, 22]. Indeed, diverse ASD-linked genes can disrupt the complex molecular circuitry that translates synaptic ion currents into intracellular signal transduction cascades, traffics those messages to sites of translation and transcription, and converts protein-level modifications into long-term changes in gene expression.

Despite these hints at convergent mechanisms, heterogeneity is still the dominant theme when comparing different autism types [23], or even when comparing genetically similar autisms. The prototypical example is the gene Shank3, responsible for Phelan-McDermid syndrome-associated autism and implicated in ~ 1% of total ASD cases [24]. Shank3 encodes multiple alternatively spliced protein variants (at least six), which each contain different combinations of protein-interaction-mediating domains. No fewer than 13 different mutant mouse lines have been reported thus far, which disrupt different exons of Shank3. While the majority of lines show deficits in social (nine lines), repetitive (nine lines), or vocalization (four lines) behavior, each line shows a different combination of behavioral and molecular deficits, depending on which Shank3 isoforms are disrupted (reviewed in [24]). For example, in a complete knockout line, *reducing* mGluR5 activity normalized repetitive grooming [25], while in an exon 11 deletion line, *enhancing* mGluR5 activity rescued abnormal grooming [26]. Similarly, at the level of electrophysiology, *reduced* striatal mEPSP amplitude and frequency has been reported in adult Shank3B^−/−^ striatum [27], reduced amplitude but increased frequency in Shank3Δex4–9^+/−^ hippocampus [28], increased mEPSP frequency and amplitude in p14 Shank3B^−/−^ striatum [29], and increased activity in p14 Shank3B^−/−^ cortex [29]. Thus, even models targeting the same gene display different phenotypes dependent on mutation type, age, and brain region. For the majority of ASD genes, only a single model (typically a complete knockout) has been published, and the ages and brain regions targeted differ between labs, complicating attempts at directly comparing pathology between published studies.

This study was designed to make a series of identical, directly comparable molecular measurements in several mouse models of autism, in order to address the question of molecular convergence among models. We compared measurements of synaptic proteins in two brain regions (frontal cortex and hippocampus), in age-and-sex-matched adult (postnatal day 60) animals from six genetic and one environmental model of ASD. We used a novel proteomic technique, Quantitative multiplex co-immunoprecipitation (QMI), that compares the abundance of, and interactions among, a panel of native proteins in mutant animals vs. a matched wildytype littermate control. In QMI, protein complexes are immunoprecipitated onto 5 um polystyrene latex beads and probed with fluorophore-coupled antibodies to quantitatively measure the amount of proteins in shared complexes. The resulting fluorescent signals are read on a flow cytometer, and raw abundance measures are normalized to wildtype controls run on the same plate to cancel out batch effects; only fold-change values compared to control are reported [30].

QMI is a candidate-based approach that targets carefully selected networks of interacting proteins. The high-dimensional data produced is linear over a large dynamic range and is several-fold more sensitive than traditional Western blotting techniques [31]. We used a previously published QMI panel that targets 16 synaptic proteins and measures 240 binary proteins in shared complexes by exposed surface epitopes (PiSCES). This panel consists of ASD-linked proteins that are known to physically interact at the synapse [32]. In each mouse model, we identified a unique combination of disrupted PiSCES, with occasional overlap of disruptions that were common to multiple models. We then clustered the data by model and brain region to reveal possible higher-level relationships among the seven animal models, and we confirmed a previously unreported molecular deficit in one model that was predicted by our clustering. Our approach has the potential to identify unexpected commonalities among genetic autisms and to suggest novel treatments based on shared molecular pathology.

## Methods

### Animal models

The specific identity of all mouse strains used is shown in Table 1. Littermate mice were co-housed in groups of 2–5 under standard laboratory conditions. At 60 days of age, mice were deeply anesthetized with isofluorane, decapitated, and brains were removed. We chose day 60 because we wanted to focus on adult animals, since the majority of animal models have been behaviorally tested as adults (see Table 1). We used two males and two females for each genotype to focus our study on robust, non-sex-dependent effects, except FMR1^−/y^ mice and controls, which were all male since FMR1 is an X-linked gene. We used frontal cortex for all models and hippocampus for some models because these two brain regions have been frequently analyzed in electrophysiology and biochemical studies of ASD models (see Table 1). For frontal cortex, the rostral 3 mm of cortex was cut with a razor blade in a metal brain mold, making sure not to include any striatal tissue in the section, and the olfactory bulb was removed; for hippocampus, bilateral hippocampi were removed with curved forceps. Tissue was frozen in liquid nitrogen and stored at − 80 until homogenization. All work was performed under an approved animal protocol at Seattle Children’s Research Institute (#15580).

**Table 1:** Animal models used in this study

Strain	Source and background	First citation	Behavioral phenotype	Synaptic proteins	Electrophysiology
Shank3B^−/−^	Jax #017688 C57BL/6J	[27]	Abnormal social behavior; excessive grooming leading to self-injury.	Reduced PSD levels of GluR2, NR2A/2B, Homer.	In striatum: reduced mEPSP frequency and amplitude; reduced population spike amplitude.
Shank3Δex4–9^+/−^	Jax #017890 C57BL/6J	[28]	Less social sniffing, fewer vocalizations in males exposed to females.	Reduced GluR1 immunofluorescent puncta in hippocampus.	In hippocampus: reduced mEPSP amplitude but increased frequency. AMPAR-dependent deficit in population spike amplitude. Impaired LTP, normal LTD.
Ube3a^2xTG^	Jax #017482 FVB	[34]	Abnormal social behavior, fewer ultrasonic vocalizations in adult animals, increased repetitive grooming	Reduced ARC levels.	In whisker barrel cortex: reduced mEPSP frequency and amplitude; reduced evoked EPSC amplitude.
Cntnap2^-/-^	Jax #017482 C57BL/6J	[46]; in ASD context [64]	Abnormal social behavior, fewer pup ultrasonic vocalizations, increased repetitive grooming		Somatosensory cortex: reduced population synchronization by Ca2+ imaging.
TSC2^+/−^	Jax #004686 B6129SF2/J	[65]	Abnormal social behavior [63], impaired hippocampal-dependent memory [37]. Abnormal pup vocalizations [66]	Hippocampus: increased mGluR5 [67].	Hippocampus: enhanced LTP [37], reduced LTD in juveniles, abnormal but present LTD in adults [67].
FMR^−/y^	Jax #003025 C57BL/6 J	[68]	Abnormal social behavior, increased repetitive behaviors, impaired memory; reviewed in [69].	Less mGluR5 in forebrain PSD preps; normal levels of other GluRs; normal levels of all receptors in total membrane lysates. [70].	Hippocampus: enhanced, abnormal LTD; LTP reported impaired or normal (reviewed by [69]). Cortex: normal LTP at 2 months, abnormal at 12 months [71].
VPA	Generated by M. Fahnestock, McMaster U. CD-1	[72] (established model); [73] (tested behavior)	Reduced social interaction, increased repetitive/stereotyped behaviors [73], reduced vocalizations [74],	Cortex of 2-week-old rats: increased NR2A/B, [35]; adult rats: reduced PSD95 [52].	Cortical pyramidal neurons: enhanced NMDAR-mediated currents, enhanced LTP [35].

VPA mice were prepared at McMaster University in compliance with standards of the Canadian Council on Animal Care and with approval from the McMaster University Animal Research Ethics Board. CD-1 female mice were mated until a sperm plug was detected (E0). On day 12.5 after conception (E12.5), pregnant mice received a single intraperitoneal (i.p.) injection of 500 mg/kg sodium valproate (VPA; Sigma, Oakville, ON, Canada) dissolved in 0.9% NaCl solution, while controls were injected with only saline. E12.5 was chosen to match previous reports from our group and others (see [32]). Pups were weaned on postnatal day (PD) 21 and subjected to behavioral assays (three-chamber sociability, elevated plus maze, and marble-burying assays for social behavior, anxiety, and repetitive behavior, respectively) on PDs 29–34. Animals were killed by decapitation on PD 35, and brains were rapidly dissected and stored at − 80 °C.

### QMI analysis

Tissue was homogenized in 0.32 M Sucrose in HEPES buffer, pH 7.4 with Sigma protease (Cat # P8340) and phosphatase (Cat # P5726) inhibitors (Sigma Aldrich), using 12 strokes of a glass-Teflon homogenizer. Samples were spun at 1000×*g* for 5 min to pellet membranes, then spun at 10,000×*g* for 15 min to pellet P2 synaptosomes. Synaptosomes were solubilized on ice in 200 ul lysis buffer (150 mM NaCl, 50 mM Tris (pH 7.4), 1% NP-40, 10 mM NaF, 2 mM sodium orthovanadate + protease/phosphatase inhibitor cocktails [Sigma]) for 15 min, spun at 4 °C at 10,000×*g* for 15 min to remove insoluble material, and protein concentration was measured by BCA assay (Thermo-Fisher).

QMI beads (Luminex) were prepared as previously described [32], with each bead color-class coupled to a distinct immunoprecipitating antibody, as shown in Table 2. Equal amounts of protein from each matched pair of animals (transgenic vs. wild type littermate or VPA- vs. saline-treated control) were incubated with QMI beads overnight at 4 °C, with constant rotation. Beads from each sample were then distributed into 32 wells of a 96-well plate, approximately 250 beads of each class per well, and each of 16 probe antibodies was added, in duplicate, to individual wells. Beads were then washed with ice-cold Fly-P buffer [50 mM Tris (pH 7.4), 100 mM NaCl, 1% bovine serum albumin, and 0.01% sodium azide], incubated for 30 min with streptavidin-PE (1:200, BioLegend), washed again, and read on a custom refrigerated Bioplex 200 flow cytometer (BioRad), which recorded the bead classification (corresponding to IP’d protein, X) and PE fluorescence (corresponding to the amount of probe antibody target protein, Y) of each bead. An above-background reading for IP:X Probe:Y indicates the occurrence of a protein complex containing both X and Y [33].

**Table 2:** QMI targets, autism linkage, and antibody information

Gene name	Protein name	Description	Simons score	Evidence in ASD	IP antibody	Probe antibody
GRM5	mGluR5	Metabotropic glutamate receptor. G-protein coupled receptor activates Erk and PI3K cascades in response to glutamate.	NS	Rare variants identified in ASD patients [61, 75]. Plays a key role in Fragile X [41] and possibly other ASD models.	Millipore 5675 Cat#AB5675	Neuromab N75/3 Cat# 75-115
GRIN1	NMDAR1	NMDA-type glutamate receptor subunits. NMDARs are heterotetramers with high Ca2+ permeability, essential for learning and memory. Subunits confer different functional properties to the receptor.	3	Rare variant in ASD siblings [76]	Thermo 54.1 Cat# 02-0500	Santa Cruz polyclonal C20 Cat# sc-1467
GRIN2A	NMDAR2A		4	Genetic association [77]	Neuromab N327/95 Cat#75-288	Biolegend N327A/38 Cat# 832401
GRIN2B	NMDAR2B		1	Multiple rare variants identified in ASD patients. [4, 78, 79]	Biolegend N59/20 Cat# 832501	Biolegend N59/36 Cat# 818701
GRIA1	GluR1	AMPA-type glutamate receptor subunits. AMPARs are also tetramers. Subunits confer different functional properties to the receptor.	2	Recurrent missence mutations [4, 80]	Biolegend N355/1 Cat# 819801	Millipore polyclonal 1504 Cat# AB1504
GRIA2	GluR2		–	Contained in an ASD-linked deletion [81]	Biolegend L21/32 Cat# 810501	Santa Cruz polyclonal C20 Cat# sc-7610
NLGN3	NL3	Postsynaptic Neuroligin 3 binds presynaptic neurexins to create trans-synaptic adhesion bridges. Cleavage following activity may be involved in cell signaling.	2	Rare mutations; TADA study [82, 83]	Thermo 566209 Cat# MA5-24253	Santa Cruz G2 Cat # sc-271880
HOMER1	Homer1	Postsynaptic scaffold linking Shanks, mGluRs, and many other postsynaptic components. Forms homo-tetramer via N-terminal coiled-coil domain.	4	Rare variants [61]	Lifespan Biosciences AT1F3 Cat# LS-C103482	Santa Cruz D3 Cat # SC-17842
	Homer1A	Activity-dependent isoform of Homer1 that lacks coiled-coil domain and acts as a dominant negative, preventing scaffolding by long Homers.			–	Santa Cruz polyclonal M13 Cat# sc-8922
DLG4	PSD95	Major component of postsynaptic density, scaffolds NMDARs and other components of the PSD via multiple binding domains.	NS	Rare variants and network gene analyses [84–86]	Biolegend K28/74 Cat# 810301	Biolegend K28/43 Cat# 810401
DLG1	SAP97	Scaffold with similar function to PSD95, but differentspecific binding affinities.	NS	Exome sequencing revealed rare variants [85]	Enzo RPI197.4 Cat# ADI-VAM-PS00	SantaCruz polyclonal H60 Cat# sc-25661
SHANK3	SHANK3	Scaffolding protein that forms a polymeric structure with Homer, and links multiple receptor types to downstream signaling pathways	1S	Recurrent rare de novo mutations and copy number variations (deletions) [87]	NeuroMab N367/62 Cat# 75-344	Enzo Life Sciences RPI197.4 Cat# ADI-VAM-PS00
UBE3A	Ube3A	E3 ubiquitin ligase, phospho-regulated by synaptic activity, that ubiquitinates Arc as well as other neuronal targets; also acts as a transcriptional regulator.	3S	Rare variants and copy number variations (duplications) [88, 89]	Santa Cruz H182 Cat# sc-25509	Santa Cruz E-4 Cat # sc-166689
SYNGAP1	SynGAP1	A RAS GTPase that is heavily expressed at the PSD. Negatively regulates RAS. PSD95 binding may be important in regulating synaptic binding “slots.”	1S	Multiple rare variants [90]	Cell Signaling D20C7 Cat# 5539	SantaCruz polyclonal R19 Cat# sc8572
FYN	Fyn Kinase	Associates with PSD95 and NMDARs, phosphorylates the latter. Also binds and is activated by mGluRs.	–	None reported	Santa Cruz Fyn15 Cat# sc-434	BioLegend FYN59 Cat# B149751
PIK3R1	PI3K	PI3K is a lipid kinase that phosphorylates membrane phospholipids and initiates PI3K/AKT/mTOR signaling. The enzyme consists of a p85 regulatory and p110 catalytic subunit. Our antibodies target p85alpha.	–	PI3K subunits PIK3CG and PIK3R2 (Simons score 4 and S, respectively) have been linked to autism [91, 92]	Thermo-Fisher U5 Cat# MA1-74183	Millipore AB6 Cat# 05-212

NS Listed in SFARI gene but not scored; − not listed

### Data analysis

**Fig. 1:**
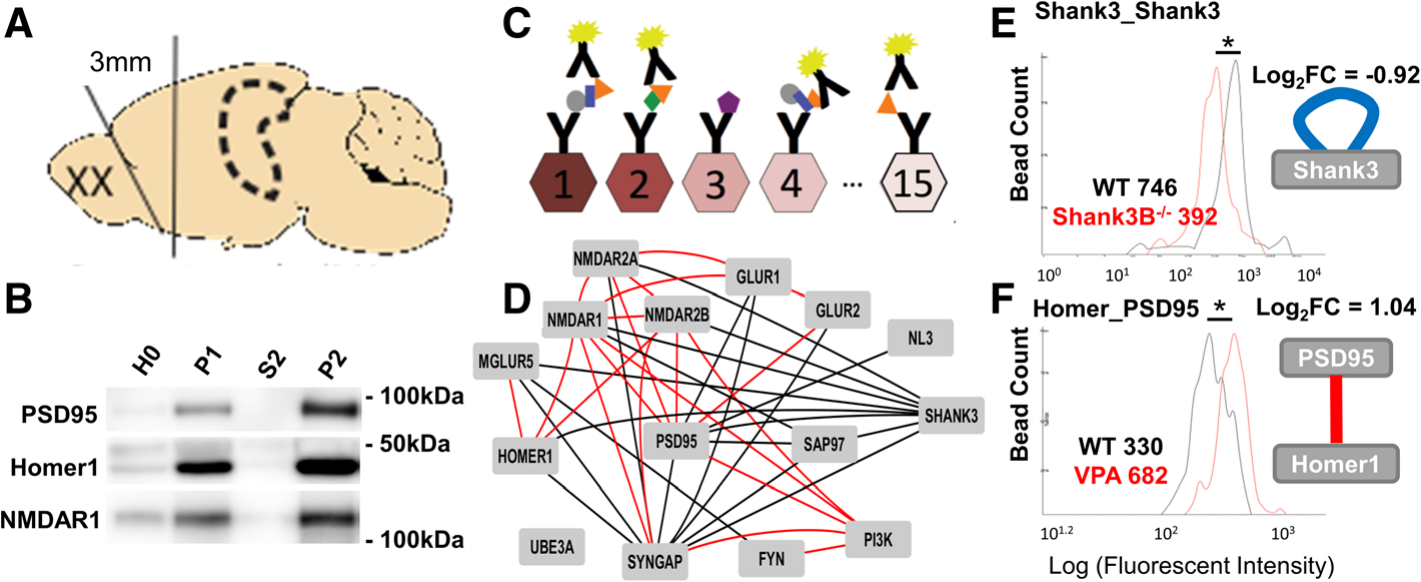
Workflow. **a** 3-mm sections of frontal cortex or bilateral hippocampi were collected from matched pairs of wildtype and mutant littermates. **b** P2 fractions were prepared to enrich for synaptic proteins. Shown here is typical enrichment of Homer1, PSD95, and NMDAR1 in P2 fractions, compared to equal amounts of total protein (by BCA assay) from brain homogenate (HO), P1 membrane pellet, and S2 soluble protein. **c** A panel of IP beads, each conjugated to a different antibody, is incubated with lysate, probed with fluorophore-conjugated antibodies, and read on a flow cytometer. **d** Known protein-protein interactions among the targeted protein network, in mouse, from the BioGRID database. Red lines indicate IP-western interactions, black lines IP-mass spectrometry. **e**, **f** Example histograms and corresponding node-edge visualizations. **e** Reduced IP: Shank3 Probe: Shank3 in a Shank3B^−/−^ animal. Blue loop on Shank3 indicates a negative log_2_FC of an IP_Probe for the same target. **f** Increased Homer_PSD95 in VPA cortex lysate (red) and matched wildtype littermate control (black). Red line between nodes indicates positive log_2_FC of an interaction

Data were exported in .xml files containing all data on a bead-by-bead, well-by-well basis. A custom Javascript was written to generate histograms showing bead distributions for a given bead class in a given well and to extract the median fluorescent intensity of each bead class in each well for export to Excel and R (faculty.washington.edu/seps/program). A custom MatLab script, “Adaptive Non-parametric statistical test with an adjustable alpha Cutoff” (abbreviated ANC), previously described in detail [30], was used to identify interactions that changed significantly in > 70% of experiments; these interactions are referred to as “hits.” ANC first uses a K-S test to compare histogram distributions of technical replicates to both discard duplicate wells that are significantly different from each other (presumed manual error) and to adjust the alpha value based on technical error. K-S test results from comparisons between an experimental sample and a matched control are then corrected for multiple comparisons and technical errors to obtain a final *p* value. “Hits” were interactions with *p* < 0.05. Please see [30] for details. Prior QMI analysis in both T cells [30] and neural tissue [32] found that N of four biological replicates are sufficient to produce a consistent number of significant hits, so an N of at least four matched pairs was used. To eliminate batch effects due to both technical and biological variation, we limit comparisons to ASD model animals and co-housed, littermate controls euthanized on the same day and run on the same assay plate; ANC statistics are therefore based on consistent differences in paired comparisons for *N* = 4 experiments (each run with technical replicates). Workflow and examples of smoothed histograms are shown in Fig. 1.

Data matrices for each matched pair were exported from Java to Excel. For each matrix position, we divided the median fluorescence value (of the two technical replicates) of each ASD model animal by its wildtype littermate control and log_2_-transformed the result. Then, log_2_ fold change (log_2_FC) matrices from *N* = 4 experiments were averaged to generate a single mean log_2_FC matrix per genotype/tissue type, shown in Additional file 1: Table S1. ANC significant hits were identified and imported into Cytoscape for visualization. Significant interactions are represented by an edge connecting two protein nodes; the color and width of the edge corresponds to the direction (red = up, blue = down) and magnitude of the change (Fig. 1e, f). Changes in protein abundance (IP probe of same target protein) are represented by loops.

To cluster samples by shared ANC-significant hits, we used the hclust(dist()) and heatmap.2 functions in R. To cluster samples by average fold-change matrices shown in Additional file 1: Table S1, we first performed principal component analysis to reduce noise due to nonspecific background fluctuations using the “PCA” function in the “FactomineR” package for R; then we used the “HCPC” function in the same package to cluster genotypes/tissue types by principal components. To test the robustness of clustering, we used the “pvclust” function in R. All options were used in the default settings.

Western blots were run on cortical tissue using standard protocols. Briefly, cortical P2 fractions were lysed in lysis buffer, protein concentrations were normalized using BCA assays, equal amounts of protein were loaded into each well and run at 110 V. Protein was transferred onto PVDF membranes, blocked with 5% milk in TBS-T, primary antibodies were incubated overnight at 4C, followed by washes, species-specific secondary antibody incubation (anti-mouse or rabbit, 1:10,000, Jackson Immunoresearch), and luminol detection (Pierce Femto reagent). Antibodies used (all 1:1000 dilutions) the following: Ube3a clone E-4 (Santa Cruz), pAKTs473 clone D9E, pAKTt308 clone 244F9, panAKT clone 40D4, pMTOR polyclonal Cat #2971, and pS6 clone D57.2.2 (all from Cell Signaling).

## Results

Additional file 1: Table S1 shows the median log_2_ fold change values for the complete dataset (*N* = 92 samples from 56 animals; 7 ASD models, with 4 ASD model animals and 4 WT controls per group, except *N* = 6 for Shank3B hippocampus; some animals contributed both cortical and hippocampal tissue). Numbers in bold case indicate fold changes > 1.19 or < 0.84 (which corresponds to ± 0.25 in log_2_ scale), while red highlighting indicates that a value was statistically significant by ANC statistical analysis. Note that while some ANC-significant values are smaller than ± 0.25, indicating a small but high-confidence change, several bolded cells are not ANC-significant due to biological or technical variation and the stringent requirements of our statistical test. Below, we first focus our analysis on only significant ANC hits, then we perform inter-model comparisons using the entire data matrix to attempt to cluster models into biologically relevant groups.

### Cortex

**Fig. 2:**
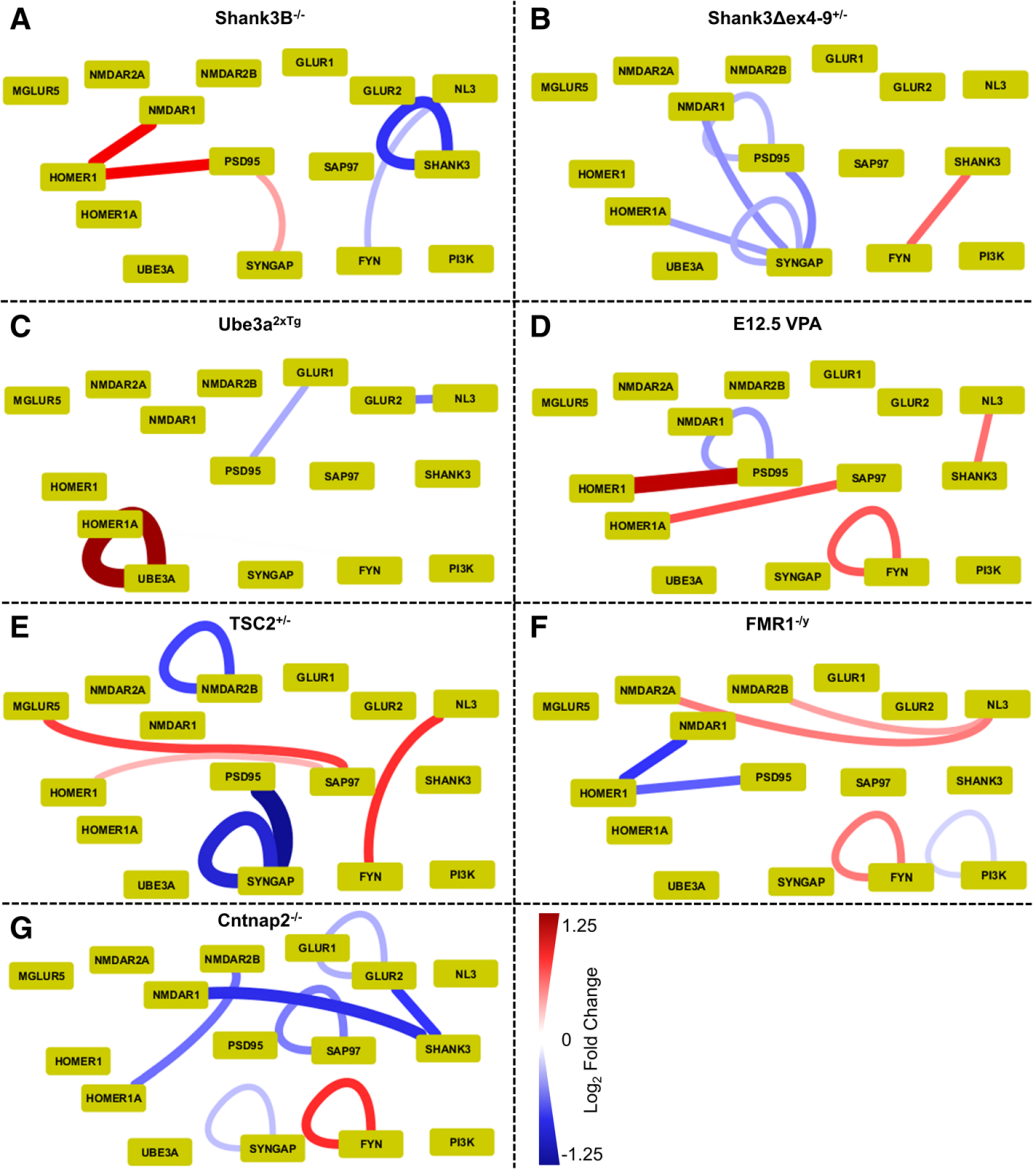
Cortical QMI Diagrams for seven autism models. Edges indicate ANC-significant (*p* < 0.05) changes in the connected nodes; red = increased, blue = decreased in mutant/wildtype comparisons. Node thickness and color indicates the magnitude of the change. **a** Shank3B^−/−^, *N* = 4 pairs. **b** Shank3Δex4–9^+/−^, *N* = 4 pairs. **c** Ube3a^2xTG^, *N* = 4 pairs. **d** E12.5 VPA exposed animals, *N* = 4 pairs. **e** TSC2^+/−^, *N* = 4 pairs. **f** FMR1^−/y^, *N* = 4 pairs. **g** Cntnap2^−/−^, *N* = 4 pairs

Overall, we found 32 statistically significant differences across the 7 mouse models (Additional file 1: Table S1 Sheet 2, and Fig. 2). Of 240 total IP_Probe combinations, 9 proteins (IP probe for the same target) and 18 proteins in shared complexes (PiSCES—IP probe for different proteins) showed differences in abundance across the 7 models. Four PiSCES (Homer1_PSD95, Homer_NMDAR1, SynGAP_PSD95 and NL3_FYN) and three abundance measures (FYN, SynGAP and PSD95) were significantly different in multiple models, while the remaining differences were unique to a single model.

*Shank3B*^*−/−*^ animals (Fig. 2a) showed a reduction in Shank3 protein levels and an increased co-association of Homer with PSD95 and NMDAR1. This is counterintuitive, since Homer-PSD interactions are likely mediated by Shank proteins, and may be expected to be reduced in Shank3 animals. These results may reflect changes in Shank1/2 vs. 3 scaffolding, or an increase in these activity-labile interactions [32] may be downstream of reduced synaptic activity in mutant animals [27]. Interestingly, these same two interactions were changed, but in the opposite direction, in FMR^−/y^ mice (see below), which have increased basal activity, potentially supporting an activity-dependent mechanism (see discussion). We also observed a small increase in SynGAP_PSD95 (also activity-labile) and a small decrease in FYN_NL3, interactions that were both observed in the opposite direction in the TSC2^+/−^ model.

*Shank3Δex4–9*^*+/−*^ animals (Fig. 2b) were tested as heterozygotes because the heterozygotes showed abnormal behavior in the original publication [28] and more accurately represent the human condition, a heterozygous deletion/mutation. Consistent with only moderately reduced Shank3 levels, a modest reduction in Shank3 (log_2_FC = − 0.227) was not significant by ANC. Remarkably, there was no overlap in significant hits with Shank3B knockouts; in fact, SynGAP_PSD95 was significant in the opposite direction in the two models. Similar to the TSC2^+/−^ animals, Shank3Δex4–9^+/−^ animals showed significant decreases in SynGAP_SynGAP and SynGAP_PSD95, in addition to reductions in SynGAP_Homer1A and SynGAP_NMDAR1 that were unique to this model. Finally, a moderate *increase* in FYN_Shank3 was observed. While published electrophysiology revealed reduced excitatory transmission in both the Shank3B^−/−^ and Shank3Δex4–9^+/−^ animals [27, 28], the experiments were performed in different brain areas (striatum and hippocampus, respectively) and showed small but important differences, such as reduced vs. increased miniature EPSP frequency, respectively. In summary, while QMI data from the Shank3Δex4–9^+/−^ animals highlight reduced SynGAP associations with NMDARs and scaffolds, the Shank3B animals show differences in Homer-PSD-NMDAR complexes but no changes in SynGAP. These data suggest that the molecular deficits in the two animal models may be quite different, consistent with the different isoforms that are affected in the two models [24].

*Ube3a*^*2xTg*^ mice (Fig. 2c) showed an expected increase in the amount of Ube3a and reduced co-association between GluR1_PSD95 and NL3_GluR2. Prior work in the cortex of Ube3a animals showed reduced glutamatergic transmission [34] and reduced scaffolding of GluRs is therefore consistent with prior observations.

*E12.5 VPA mice* (Fig. 2d) are the only non-genetic model analyzed here. Mice were generated by injection of VPA on E12.5, and the efficacy of the treatment was confirmed by behavioral testing (as in [32]) of the adult offspring before dissection and QMI analysis. We observed a large increase in the amount of co-associated Homer_PSD95. In all other models, the amount of Homer_NMDAR1 correlated with Homer_PSD95, and VPA mice were trending towards an increase in this interaction as well (log_2_FC = 0.48, NS). In addition, levels of Fyn were increased, PSD95 were decreased, and interactions between SAP97_Homer1A and Shank3_NL3 were increased. Prior reports in VPA-treated rat cortex showed enhanced NMDAR-mediated synaptic currents and enhanced LTP [35], consistent with the observed increase in Homer-NMDAR scaffolding. Decreased levels of PSD95 have also been reported by Western blotting [52].

*TSC2*^*+/−*^ mice (Fig. 2e) showed large reductions in the abundance of SynGAP and SynGAP_PSD95. The mTOR activator Rheb (ras homolog enriched in brain), which is directly suppressed by the TSC1/2 complex, is activated by SynGAP following NMDAR stimulation [36], so the reduction of SynGAP may be a homeostatic response to chronically activated Rheb. Reduced levels of NMDAR2B were also observed, along with increased abundance of complexes containing SAP97_mGluR5, Homer1_SAP97, and Fyn_NL3. Taken together, these data indicate reduced NMDAR2B and SynGAP expression, abnormal scaffolding of mGluR5 to Sap97, and abnormal FYN signaling, which could contribute to the altered LTP phenotype reported in the hippocampus of TSC mice [37].

*Fragile X* mice (Fig. 2f) showed increased abundance of complexes containing NMDAR2A_NL3 and NMDAR2B_NL3. Both NMDA receptors [38] and Neuroligins [39] bind PSD95, which could mediate this observed interaction. FragileX mice also showed reduced complexes with Homer1_PSD95 and Homer1_NMDAR1, demonstrating disrupted Homer-Shank-PSD95-NMDAR complexes, consistent with previous reports [40–42]. These activity-dependent interactions were also significant hits in the Shank3B and E12.5 VPA models, but in the opposite direction, possibly reflecting hyper- vs. hypo-activity of cortical neurons in these models. Finally, reduced levels of PI3K and increased Fyn were detected, consistent with disrupted kinase cascades downstream of mGluR5 in FMR1 mice [43–45].

*CNTNAP 2 KO* mice (Fig. 2g) showed the greatest number of ANC hits (7) as well as many large but non-significant changes. The abundance of GluR2 was reduced, accompanied by reduced GluR2_Shank3, NMDAR1_Shank3, and NMDAR2B_Homer1A, consistent with reduced scaffolding and expression of glutamate receptors. In addition, the detected levels of Sap97 and SynGAP were reduced, while Fyn was increased, a change also observed in the Fragile X model. While the CNTNAP2 gene product CASPR is known to cluster at the nodes of Ranvier following myelination [46], acute CASPR knockdown acts cell-autonomously to reduce both AMPA and NMDA-mediated EPSPs [47], congruent with reduced NMDAR and AMPAR levels and scaffolding observed here.

### Hippocampus

**Fig. 3:**
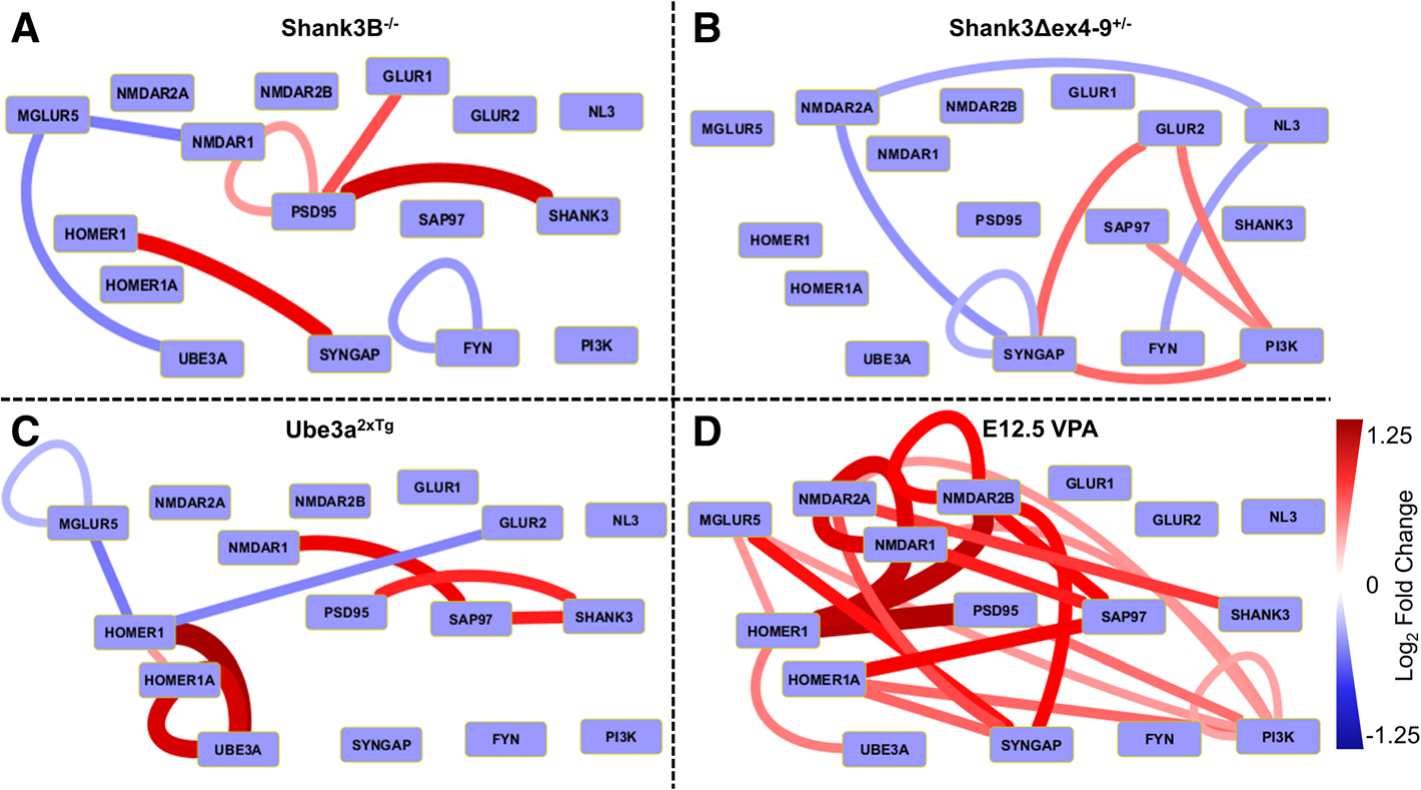
Hippocampal QMI diagrams for four autism models. Edges indicate ANC-significant (*p* < 0.05) changes in the connected nodes; red = increased, blue = decreased in mutant/wildtype comparisons. Node thickness and color indicates the magnitude of the change. **a** Shank3B^−/−^, *N* = 6 pairs. **b** Shank3Δex4–9^+/−^, *N* = 4 pairs. **c** Ube3a^2xTG^, *N* = 4 pairs. **d** E12.5 VPA exposed animals, *N* = 4 pairs

In four models, we also isolated P2 fractions from the hippocampi of the same animals that supplied cortical tissue. We identified 45 statistically significant differences across the 4 mouse models, with the majority of differences, 21, found in the VPA hippocampus (Fig. 3). Nine proteins showed differences in protein abundance (IP probe for the same target), and 32 protein interactions showed differences (IP probe for different targets). However, only three complexes (Homer_mGluR5, Sap97_NMDAR1, and SynGAP_NMDAR2A) and 1 abundance measure (PSD95) were detected in multiple models, while the remainder was unique to a single model. Below, we describe the findings from each model, compared with prior data from the cortex of the same model.

*Shank3B*^*−/−*^ hippocampal tissue (Fig. 3a) showed an increase in PSD95 levels and a decrease in FYN levels, neither of which were observed in cortical tissue. A decrease in NMDAR1_mGluR5 likely reflects disrupted scaffolding linking the two receptor types via PSD95/Shank3/Homer linkages [24]. We observed an increase in Homer1_SYNGAP, PSD95_GluR1, and, counter-intuitively, PSD95_Shank3. The latter interaction may reflect elevated expression of an alternative isoform of Shank3 that lacks the PDZ domains in complex with PSD95, possibly mediated via another protein such as Homer. Besides this interaction, no major changes in Shank3 were detected, likely due to the fact that very little Shank3 was detected from Shank3 IPs or probes, consistent with low hippocampal Shank3 expression. We are unable to relate these changes to known electrophysiological abnormalities in these animals, since to our knowledge, hippocampal electrophysiology has not been reported in this model.

*Shank3Δex4–9*^*+/−*^ animals (Fig. 3b) showed reduced levels of SynGAP, consistent with cortical tissue from these animals. Interactions involving SynGAP_NMDAR2A were reduced, while SynGAP_GluR2 were increased. Complexes containing NL3_NMDAR2A and _FYN were reduced. Complexes containing PI3K_Sap97, _GluR2, and _SynGAP were all increased. Hippocampal electrophysiology in this model indicated reduced basal AMPA-mediated transmission, and a failure of hippocampal LTP that was correlated with failure to maintain spine expansion following a tetanizing stimulation [28]. Our results indicate that SynGAP, a critical mediator of signal transduction downstream of NMDARs [36, 48], is dysregulated in hippocampal tissue prior to any type of stimulation. Further, changes in FYN and PI3K suggest downstream disruption of signaling cascades.

*Ube3a*^*2xTG*^ hippocampus (Fig. 3c) showed the expected increase in Ube3a expression, the only change that was consistent between hippocampus and cortex. A reduction in mGluR5 levels, Homer_mGluR5, and Homer_GlurR2 suggest reduced Homer-mediated scaffolding. Ube3A_Homer interactions were strongly increased, although the significance of this increase is unclear since Ube3a has not been documented to bind directly to or ubiquinate/degrade Homer proteins. The amount of PSD95_Shank3 was increased, as was SAP97_NMDAR1 and SAP97_Shank3. Finally, the amount of Homer1A was increased. These data demonstrate complex changes in scaffolding of AMPA, NMDA, and metabotropic glutamate receptors mediated by both Homer and DLG scaffolds in the Ube3a^2xTG^ animal. Hippocampal electrophysiology has not been reported in these animals, although LTP disruptions due to lack of small conductance potassium channel 2 (SK2) channel regulation have been reported in the Ube3a knockout animal [49].

*VPA hippocampus* yielded 21 significant QMI hits, the most of any sample tested, all in the positive direction. PI3K was involved in six significant interactions, with _mGluR5, _NMDAR1, _NMDAR2A, _PSD95, _HOMER1A, and _PI3K. These disruptions in PI3K, which controls AKT/mTOR signaling, is consistent with several reports implicating dysregulated mTOR signaling in the VPA model [32]. The amount of Homer_PSD95, Homer_NMDAR2B, and Homer_NMDAR1 were each increased by almost twofold, reflecting increased NMDAR scaffolding and/or expression. Levels of detected NMDAR1 and NMDAR2B were also increased. These data support prior studies showing increased NMDAR expression in rats following VPA exposure in the cortex [35], although note that a separate study did not find differences in mRNA expression in the cortex or hippocampus [50]. Other notable hits included SynGAP_NMDAR2A and B, SAP97_NMDAR1 and 2B, and Homer_mGLUR5. Comparing these results with VPA cortex, only 2/5 QMI hits in the cortex were shared with the hippocampus, Homer_PSD95 and SAP97_HOMER1A. However, several other interactions that were significant in hippocampus were trending towards significance in cortex; for example, Homer_NMDAR1 and _NMDAR2B were increased by 1.29 and 1.37-fold in the cortex, respectively, but were not significant by ANC criteria (see Additional file 1: Table S1).

#### Comparisons between models

**Fig. 4:**
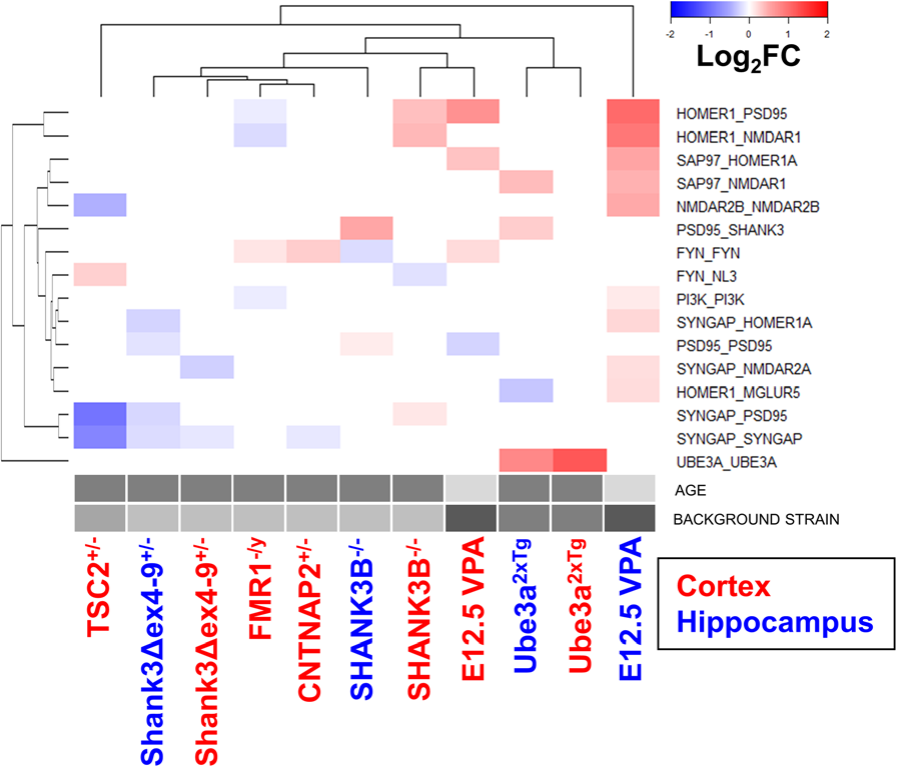
Summary of ANC-significant interactions present ≥ 2 models. Columns are clustered by genotype/tissue type, while rows are clustered by each protein interaction/abundance measure. While this format is useful to give an overview of shared ANC hits, so few hits are shared by multiple models that clustering occurs based on only 1–2 common hits, making the clustering unreliable. Model identifiers in blue represent hippocampal tissue, red cortical tissue. Gray bars indicate the potential confounding factors of age and background strain (see Table 2)

For the most stringent possible clustering analysis between models, we set all non-ANC-significant measurements to 0 and performed unsupervised clustering using the “complete” method, based on the Euclidian distance matrix of all samples. Because interactions that were significant in a single sample are irrelevant for clustering using this method, we only included the 16 interactions that were significant in two or more samples (Fig. 4). The plot highlights the correlation between certain interactions, such as Homer_PSD95 and Homer_NMDAR1, or SynGAP_PSD95 and SynGAP_SynGAP. However, it is clear from this plot that because there were relatively few interactions that reached ANC significance in multiple models, the clustering is not robust; for example, FragileX and CNTNAP2 mice are shown associated with each other on the basis of a single shared ANC-significant hit, Fyn_Fyn.

**Fig. 5:**
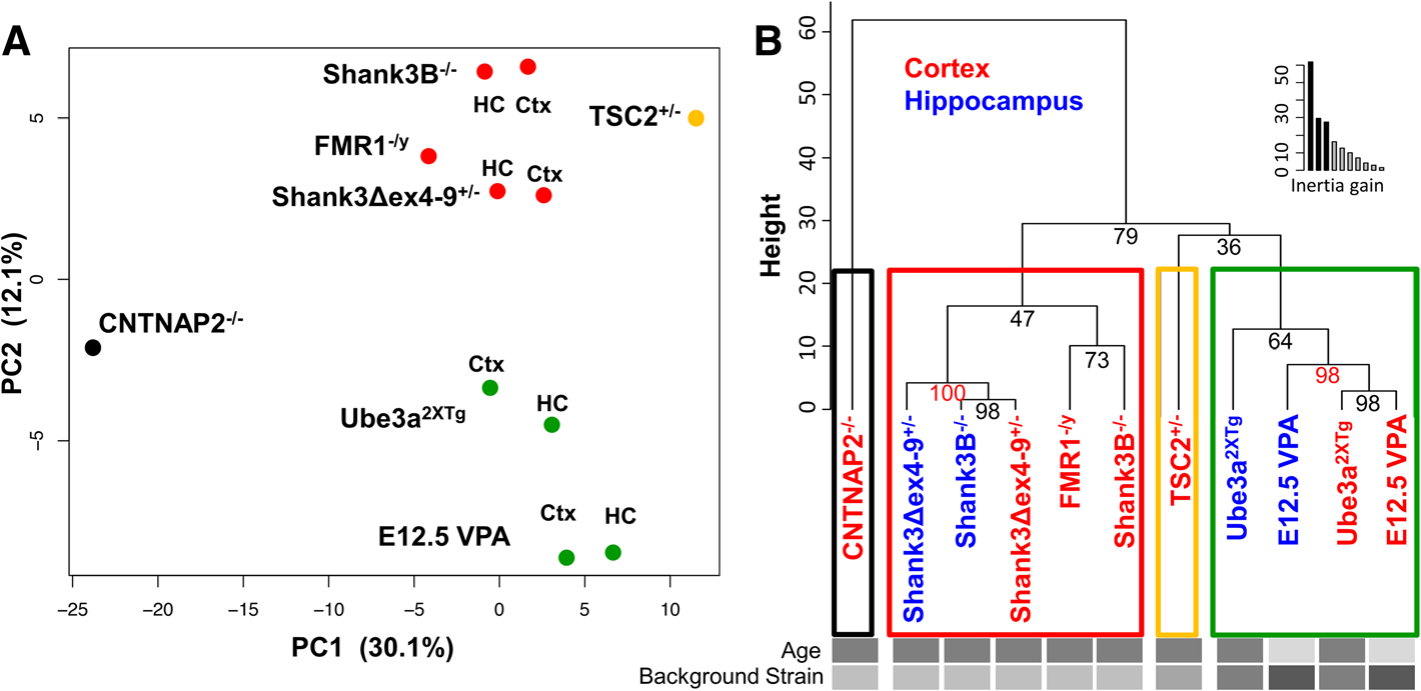
Clustering models by log_2_FC matrices. **a** Principal component analysis of all genotypes/tissue types. Each group is plotted by its PC1 and PC2 values. Points are colored by HCPC clustering show in B. **b** HCPC clustering of ASD models. Based on the inertia gained by cutting at each level (inset graph), the HCPC program suggested clustering into four groups as shown. Numbers at the branch points show the approximately unbiased (AU) *p* value calculated by multiscale bootstrap resampling; clusters with AU greater than 95 are strongly supported by the data. Model identifiers in blue represent hippocampal tissue, red cortical tissue. Gray bars indicate the potential confounding factors of age and background strain (see Table 2)

To overcome this limitation, we repeated our cluster analysis with all log_2_FC data, reasoning that smaller changes that did not reach the high bar for ANC significance could still be informative for clustering analysis. However, we were concerned about noise contributed by interactions that did not change, but fluctuated randomly around 0, so we first performed principal component analysis (PCA) to focus on factors that contributed the most variation to the dataset. PCA was performed on the mean log_2_FC values of each interaction for all genotypes/tissue types using default settings. Plotting the data by principal components 1 and 2, which accounted 30.1% and 12.1% of total variation, respectively (Fig. 5a), revealed clear clustering of tissue types within models; in all cases, the hippocampal and cortical tissue shared similar coordinates in PCA space. Both Shank3 models and Fragile X animals were in close proximity in PCA space, and Ube3a^2xTG^ were near VPA animals. To mathematically determine the relationships between models in PCA space, we used a hierarchical clustering on principal component (HCPC) analysis using default settings in the FactoMineR package and cutting the HCPC tree at the recommended level to maximize inertia gain (Fig. 5b). HCPC yielded four clusters: CNTNAP animals were an outgroup (group1). Group 2 contained all Shank3 models, and FMR1 animals. TSC2 animals, alone in group 3, were clustered on a branch adjacent to group 4, which contained cortical and hippocampal tissue from both VPA and Ube3a models. We calculated approximately unbiased *p* values for the clustering based on multiscale bootstrap resampling. The co-clustering of Shank3B hippocampus with Shank3Δex4–9^+/−^ tissues, and the clustering of VPA tissue with Ube3a cortical tissue reached statistical significance (AU < 0.95); AU values for all other branches are shown in Fig. 5b.

#### Shared molecular pathology in cluster 4

**Fig. 6:**
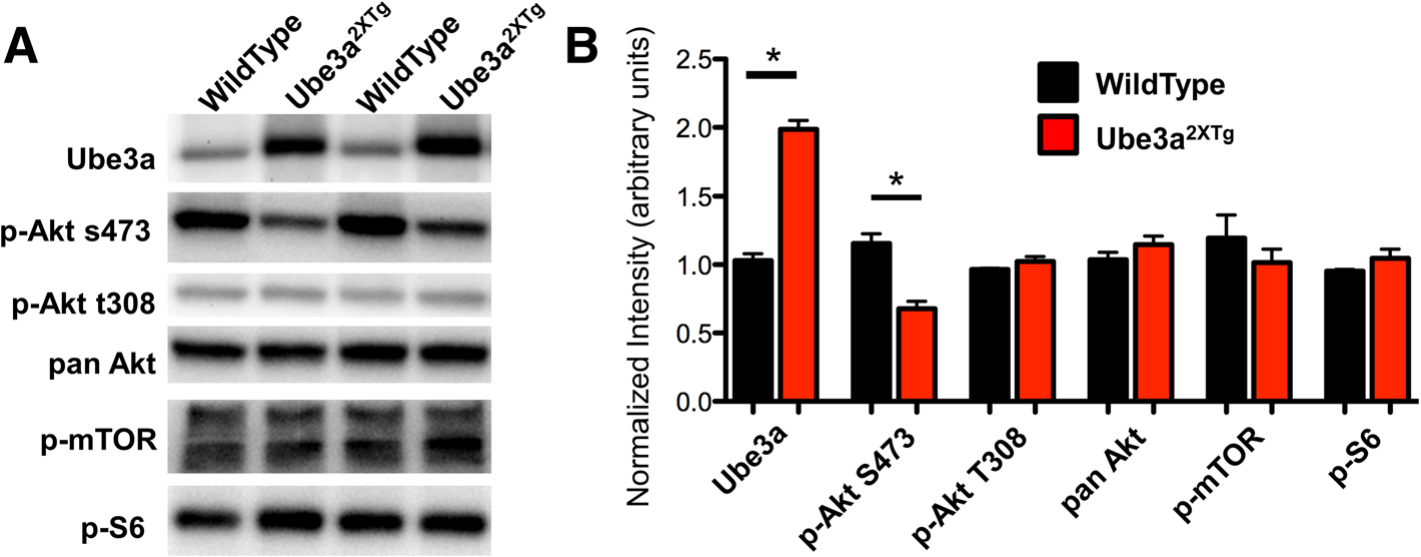
AKT phosphorylation is reduced in Ube3a^2xTG^ mice. **a** Representative western blots of synaptosomal fractions from adult mice probed with the indicated antibodies and **b** quantification. *N* (WT, Ube3a^2xTG^) = 5, 6 for all blots except 11, 12 for p-AKTs473 and panAKT. *p < 0.0001 by two-tailed *t* test

We noticed from the structure of the clustering that groups 3 and 4 contained two models with known abnormalities in the AKT/mTOR signaling pathway; in fact, mTOR inhibitors have been reported to rescue behavioral deficits in both models [37, 51]. TSC2^+/−^ mice are heterozygous for a critical inhibitor of the mTOR complex and show sustained mTOR activation and abnormalities throughout the pathway [37]. VPA animals also show abnormal AKT signaling, with a recent report showing reduced levels of AKT and mTOR, as well as reduced ratios of phospho-to-total AKT and mTOR in VPA exposed rats [52]. Ube3a^2xTG^ mice clustered closely with VPA mice, but AKT/mTOR has never been implicated in this model. Indeed, mining the factors that differentiated HCPC clusters indicated that PI3K was a significant factor that differentiated group 4, and Ube3a hippocampal issue showed a large, but non-ANC-significant increase in PI3K_PI3K (log_2_FC = 0.42, NS). We therefore performed phospho-Western blots on cortical samples from an independent cohort of Ube3a^2xTG^ animals (Fig. 6). AKT phosphorylation was reduced by 41% at p-Ser473, while no difference was observed at p-Thr308. Total AKT levels were similar. Downstream of AKT, mTOR phosphorylation was also similar, as were levels of p~S6. We confirmed previous reports of altered p-AKT levels in cortical tissue from VPA animals and found that p-AKT and total AKT levels were normal in cortical tissue from all other models examined (Additional file 2: Figure S1). These data confirm the predictions of our clustering that Ube3a^2xTG^ mice share a core deficit in the PI3K/AKT/mTOR pathway with the VPA mice that share the same branch of the HCPC cluster tree.

## Discussion

The goal of these experiments was to perform a series of identical protein measurements of brain tissue from multiple mouse models of autism, with the aim of cutting through the immense heterogeneity of the diagnostic entity and identifying some underlying points of convergence. We did not expect every animal model to show an identical set of protein network disturbances. Rather, we hypothesized that a set of interactions might be disrupted in more than one model; perhaps we would be able to identify subtypes of genetic autisms that share distinct sets of disrupted interactions. Indeed, our stringent ANC criteria identified several interactions that were common to multiple genetic models, but clustering by only ANC-significant interactions was not robust. Bioinformatics analysis using PCA and HCPC clustered the models by both genotype and tissue type indicated generally similar changes in cortical and hippocampal tissue from the same models. For such a clustering approach to be broadly useful, it would need to make testable predictions about pathologic mechanisms. Indeed, analysis of the interactions that contributed to clustering suggested that Ube3a^2xTG^ mice might share a molecular deficit with the other models sharing its branch of the tree, namely disrupted AKT/mTOR signaling. Western blots revealed that AKT signaling was disrupted in Ube3a^2xTG^ and VPA mice, but not other models, confirming clustering predictions. We have therefore successfully identified a set of protein-protein interactions that are disrupted in multiple animal models of autism, clustered models based on high-dimensional QMI data, and used our clusters to make testable predictions about the molecular pathology of closely clustered models.

Proteins or protein interactions that were ANC-significant in multiple models identified here share striking similarity to a set of interactions that we recently reported to be activity-dependent. In response to 5 min of acute stimulation with glutamate, QMI identified significant changes in 26 protein-protein interactions [﻿32﻿]. Homer, Shank and SynGAP were the most connected nodes, each changing its interactions with several other members of the network. Many of these activity-dependent interactions were also identified here as significantly different in ASD models vs. wildtype controls. For example, Homer1_PSD95 and the abundance of the Ras GTPase SYNGAP were each altered in four sample types, and interactions between Homer1_NMDAR1 and SYNGAP_PSD95 were each altered in three sample types. Of the 26 Glutamate-significant interactions, only 15 were included in the QMI panel presented in this paper; but of those 15 interactions, 9 were “hits” in the ASD models (Additional file 1: Table S1, sheet 2). Notably, the directionality of these changes was variable. For example, Homer_PSD95 levels were increased in VPA cortex, but decreased in Fragile X cortex.

Differences in synaptic activity are a defining and unifying characteristic of animal models of autism—virtually, every report of an autism model includes electrophysiological characterization showing altered synaptic transmission. The directionality of change in synaptic activity is also variable between models; for example Ube3a^2xTG^ mice show reduced cortical excitability [32] while Fragile X [53, 54] mice show increased. Viewed through this activity-dependent lens, the bidirectionality of our data makes more sense. Glutamate stimulation results in dissociation of Homer-PSD95 complexes [﻿32﻿]; thus, the reduced amount of this interaction seen in the fragile X model could reflect the hyperactive tonic signaling that has been previously reported [53, 54]. Conversely, in the VPA model, a reduction in intrinsic cortical activity has been reported [55, 56], which would be predicted to cause increased levels of this activity-labile interaction. Future studies could manipulate activity in ASD models and measure the resulting QMI profiles to directly test this hypothesis and disentangle activity-dependent from activity-independent processes.

However, activity-dependent interactions were not uniformly altered within models; for example, while Homer1_PSD95 and Homer1_NMDAR1 were reduced by activity and increased in the VPA model, SynGAP_PSD95 was also reduced by activity [﻿32], but unchanged in the VPA model. This could imply an underlying dysregulation in the network response to activity, or a de-coupling of normally correlated molecular processes due to differences in the cell’s ability to compensate for some long-term changes better than others. An analogous network-level dysregulation has been observed in transcriptomic analysis of postmortem autism brain tissue, where individual mRNAs show normal levels of abundance, but the coordinated expression of mRNAs is dysregulated, reflecting disrupted regulatory mechanisms [18]. In the future, it will be informative to design experiments that can de-couple acute, activity-dependent changes from long-term, genotype-dependent changes in PPI networks. The stimulus-dependent dynamics of protein interaction networks encode cellular information, such that different cellular inputs lead to different rearrangements of the interactome, encoding different cellular responses [57]. Understanding how information processing through this synaptic network differs in ASD models could lead to further insights into disease pathogenesis.

To our knowledge, only one other study has attempted to subtype a large number of mouse models of autism [58]. This MRI-based study found great heterogeneity in the relative size of many brain areas in ASD models vs. matched controls, but was able to identify clusters of animal models that shared similar patterns of changes. Three models were analyzed by both the current study and the Ellegood et al. study, FMR1, CNTNAP2, and Shank3B. Both Ellesgood et al. and our study clustered the FMR1 and Shank3B mice as neighbors on the same branch of the dendrogram, suggesting both structural and molecular convergence between the two models. Indeed, prior work has shown that Shank3 mRNA is posttranscriptionally regulated by FMR1 [59], and that FMR1 mice show deficits in mGluR signaling [25, 26] that is mediated by Homer and Shank-containing scaffolds [42]. More generally, genetic studies [60, 61] have implicated several genes or gene regulatory networks related to mGluR signaling in autism. It is plausible that our “cluster 2” may represent a subtype encompassing Shank3 and Fragile X models, previously and independently identified in the Ellesgood study. However, the models may have co-clustered in both studies by chance.

The PI3K/AKT/mTOR pathway has also been implicated in many diverse models of ASD [14, 52, 62], so it was noteworthy that two models with known disruptions to the mTOR pathway appeared together in clusters 3/4. TSC2 is directly involved in regulating mTORC1 downstream of AKT, and autism-linked mutations in TSC2 cause increased mTOR activation and a de-coupling of mTOR from AKT [63]. Prenatal VPA exposure causes reduced mTOR pathway protein expression and phosphorylation [52], which we confirmed here (Additional file 2: Figure S1). After our clustering results suggested a potential mTOR deficit in Ube3a^2xTG^ animals, we found by phospho-Western blots that Ube3a^2xTG^ animals showed reduced AKT S473 phosphorylation, but normal levels of T308 phosphorylation and normal phosphorylation of other components of the mTOR pathway. Rapamycin treatment has been shown to rescue behavior in both TSC2 and VPA models [51, 63], and future work could explore if correction of AKT phosphorylation in the Ube3a model might similarly correct behavioral deficits.

### Limitations

Several limitations of our study should be noted. The background strain was different among several of the models, the age of the VPA mice was different from the other six models, and mice of both sexes were used. Our analysis approach, in which each mutant animal was normalized to a matched littermate control, was designed to cancel out these effects, as well as assay-dependent batch effects, to identify differences caused by each mutation. However, this experimental design prevents us from making wildtype-to-wildtype comparisons (since batch effects cannot be normalized for mice run on different assay plates), so we are unable to unambiguously demonstrate that our clustering was not driven by uncorrected effects stemming from these differences in background, age, or sex. QMI is a candidate-based approach and shares limitations with all antibody-based assays, including potential antibody cross-reactivity and issues of binding epitope access in native protein complexes. The absence of a detected interaction cannot be interpreted as unambiguously indicating that the interaction does not exist in vivo, since occlusion of binding sites could lead to false negative results. We carefully selected and screened all antibodies used in the QMI panel [32], but antibody caveats are unavoidable. We used NP40 detergent after pilot data showed that it produced higher mean matrix MFIs than TritonX100, Digitonin, or Deoxycholate [32]. However, NP40 does not fully solubilize the core postsynaptic density, where several of our protein targets are enriched (discussed in [32]). Since detergents that solubilize the PSD also disrupt protein interactions, detergent selection is necessarily a trade-off, and further studies could more thoroughly quantify differences in synaptic QMI networks due to different detergent conditions. Finally, many of our interactions vary with neuronal activity or by brain region. Small variations in microdissection (e.g., inclusion of small amounts of striatal tissue in cortical samples) or euthanasia protocols (i.e., animal sleep/wake state prior to euthanasia) could have large effects on protein detection (e.g., Shank3, which is highly expressed in striatum, or PSD95_SynGAP, which is activity-dependent). While we were careful to perform our dissections as consistently as possible, at a similar time of day and using metal brain molds to ensure consistent slicing, thinner vibratome slicing followed by a period of controlled slice recovery in ACSF, as for electrophysiology, may be a more optimal experimental strategy to ensure normalization of both activity and location.

## Conclusions

In conclusion, we performed a series of identical QMI experiments to measure differences in the abundance of, and binary interactions among, 16 synaptic proteins in 7 mouse models of autism. Employing a mutant-littermate control design, we found a unique combination of disrupted protein interactions in each model and tissue type measured. Many of the disrupted interactions were identified as activity-dependent interactions in a separate study, highlighting the complex relationships between ASD risk genes and activity-dependent homeostatic processes [21]. PCA and cluster analysis of models revealed two identifiable sub-groups, with VPA and TSC2 mice comprising a hypothetical “mTOR” cluster, and Shank3 and FragileX mice comprising a second cluster; the latter co-clustering was consistent with a prior MRI study [58]. The inclusion of Ube3a^2xTG^ mice in the mTOR cluster led to our identification of AKT phosphorylation deficits in this model. Our data highlight the heterogeneity of ASD models, while offering hope that high-dimensional measures of biologically relevant molecular processes may allow differentiation of subtypes of ASD amenable to common treatment strategies. Future work to expand the number of ASD models analyzed and to perform similar QMI experiments in human iPS-derived neurons could offer further insights into the complex relationships among autism risk factors.

## Additional files

Additional file 1: **Table S1.** Sheet 1: Log_2_ fold change (log_2_FC) matrices from *N* = 4 experiments were averaged to generate a single mean log_2_FC matrix per genotype/tissue type. Cells highlighed in red are ANC-significant, numbers in bold case indicate a fold change greater than 0.25 or less than -0.25. Sheet 2: All hits are sorted by the number of times they appeared as ANC-significant. A red-shaded "1" indicates an ANC-significant hit in a particular model. If a hit was also significant following 5 minutes of glutamate stimulation [32], "yes" is entered into the "Glutamate hit" column. (XLSX 46 kb)

Additional file 2: ** Figure S1.** AKT phosphorylation is reduced in VPA mice but normal in all other models examined. Related to Fig. 6 (A) Representative western blots of cortical samples from adult mice probed with the indicated antibodies. (B) Quantification, axes match Fig. 6. *N* = 3–10 individuals per genotype. *p < 0.001 by two-tailed *t* test, Bonferroni-corrected for multiple comparisons. (PDF 194 kb)

## Abbreviations

ASD: Autism spectrum disorder
PiSCES: Proteins in shared complexes
QMI: Quantitative multiplex co-immunoprecipitation
VPA: Valproic acid

## References

[1] Baio J, Wiggins L, Christensen DL, Maenner MJ, Daniels J, Warren Z, Kurzius-Spencer M, Zahorodny W, Robinson Rosenberg C, White T (2018). Prevalence of autism Spectrum disorder among children aged 8 years - autism and developmental disabilities monitoring network, 11 sites, United States, 2014. MMWR Surveill Summ.

[2] O’Roak BJ, Deriziotis P, Lee C, Vives L, Schwartz JJ, Girirajan S, Karakoc E, MacKenzie AP, Ng SB, Baker C (2011). Exome sequencing in sporadic autism spectrum disorders identifies severe de novo mutations. Nat Genet.

[3] Iossifov I, O'Roak BJ, Sanders SJ, Ronemus M, Krumm N, Levy D, Stessman HA, Witherspoon KT, Vives L, Patterson KE (2014). The contribution of de novo coding mutations to autism spectrum disorder. Nature.

[4] De Rubeis S, He X, Goldberg AP, Poultney CS, Samocha K, Cicek AE, Kou Y, Liu L, Fromer M, Walker S (2014). Synaptic, transcriptional and chromatin genes disrupted in autism. Nature.

[5] Patterson PH (2011). Maternal infection and immune involvement in autism. Trends Mol Med.

[6] Edmiston E, Ashwood P, Van de Water J (2017). Autoimmunity, autoantibodies, and autism spectrum disorder. Biol Psychiatry.

[7] Shelton JF, Geraghty EM, Tancredi DJ, Delwiche LD, Schmidt RJ, Ritz B, Hansen RL, Hertz-Picciotto I (2014). Neurodevelopmental disorders and prenatal residential proximity to agricultural pesticides: the CHARGE study. Environ Health Perspect.

[8] Weiner DJ, Wigdor EM, Ripke S, Walters RK, Kosmicki JA, Grove J, Samocha KE, Goldstein JI, Okbay A, Bybjerg-Grauholm J (2017). Polygenic transmission disequilibrium confirms that common and rare variation act additively to create risk for autism spectrum disorders. Nat Genet.

[9] Grzadzinski R, Huerta M, Lord C (2013). DSM-5 and autism spectrum disorders (ASDs): an opportunity for identifying ASD subtypes. Mol Autism.

[10] Donovan AP, Basson MA (2017). The neuroanatomy of autism - a developmental perspective. J Anat.

[11] Fernández Marta, Mollinedo-Gajate Irene, Peñagarikano Olga (2018). Neural Circuits for Social Cognition: Implications for Autism. Neuroscience.

[12] Parikshak NN, Gandal MJ, Geschwind DH (2015). Systems biology and gene networks in neurodevelopmental and neurodegenerative disorders. Nat Rev Genet.

[13] Hormozdiari F, Penn O, Borenstein E, Eichler EE (2015). The discovery of integrated gene networks for autism and related disorders. Genome Res.

[14] Huber KM, Klann E, Costa-Mattioli M, Zukin RS (2015). Dysregulation of mammalian target of rapamycin signaling in mouse models of autism. J Neurosci.

[15] Estes ML, McAllister AK (2015). Immune mediators in the brain and peripheral tissues in autism spectrum disorder. Nat Rev Neurosci.

[16] Ashwood P, Krakowiak P, Hertz-Picciotto I, Hansen R, Pessah I, Van de Water J (2011). Elevated plasma cytokines in autism spectrum disorders provide evidence of immune dysfunction and are associated with impaired behavioral outcome. Brain Behav Immun.

[17] Vargas DL, Nascimbene C, Krishnan C, Zimmerman AW, Pardo CA (2005). Neuroglial activation and neuroinflammation in the brain of patients with autism. Ann Neurol.

[18] Voineagu I, Wang X, Johnston P, Lowe JK, Tian Y, Horvath S, Mill J, Cantor RM, Blencowe BJ, Geschwind DH (2011). Transcriptomic analysis of autistic brain reveals convergent molecular pathology. Nature.

[19] Pardo CA, Farmer CA, Thurm A, Shebl FM, Ilieva J, Kalra S, Swedo S (2017). Serum and cerebrospinal fluid immune mediators in children with autistic disorder: a longitudinal study. Mol Autism.

[20] Zoghbi H. Y., Bear M. F. (2012). Synaptic Dysfunction in Neurodevelopmental Disorders Associated with Autism and Intellectual Disabilities. Cold Spring Harbor Perspectives in Biology.

[21] Mullins C, Fishell G, Tsien RW (2016). Unifying views of autism spectrum disorders: a consideration of autoregulatory feedback loops. Neuron.

[22] Bourgeron T (2015). From the genetic architecture to synaptic plasticity in autism spectrum disorder. Nat Rev Neurosci.

[23] Masi A, DeMayo MM, Glozier N, Guastella AJ (2017). An overview of autism spectrum disorder, heterogeneity and treatment options. Neurosci Bull.

[24] Monteiro P, Feng G (2017). SHANK proteins: roles at the synapse and in autism spectrum disorder. Nat Rev Neurosci.

[25] Wang X, Bey AL, Katz BM, Badea A, Kim N, David LK, Duffney LJ, Kumar S, Mague SD, Hulbert SW (2016). Altered mGluR5-Homer scaffolds and corticostriatal connectivity in a Shank3 complete knockout model of autism. Nat Commun.

[26] Vicidomini C, Ponzoni L, Lim D, Schmeisser MJ, Reim D, Morello N, Orellana D, Tozzi A, Durante V, Scalmani P (2017). Pharmacological enhancement of mGlu5 receptors rescues behavioral deficits in SHANK3 knock-out mice. Mol Psychiatry.

[27] Peca J, Feliciano C, Ting JT, Wang W, Wells MF, Venkatraman TN, Lascola CD, Fu Z, Feng G (2011). Shank3 mutant mice display autistic-like behaviours and striatal dysfunction. Nature.

[28] Bozdagi O, Sakurai T, Papapetrou D, Wang X, Dickstein DL, Takahashi N, Kajiwara Y, Yang M, Katz AM, Scattoni ML (2010). Haploinsufficiency of the autism-associated Shank3 gene leads to deficits in synaptic function, social interaction, and social communication. Mol Autism.

[29] Peixoto RT, Wang W, Croney DM, Kozorovitskiy Y, Sabatini BL (2016). Early hyperactivity and precocious maturation of corticostriatal circuits in Shank3B(−/−) mice. Nat Neurosci.

[30] Smith SE, Neier SC, Reed BK, Davis TR, Sinnwell JP, Eckel-Passow JE, Sciallis GF, Wieland CN, Torgerson RR, Gil D (2016). Multiplex matrix network analysis of protein complexes in the human TCR signalosome. Sci Signal.

[31] Smith SE, Bida AT, Davis TR, Sicotte H, Patterson SE, Gil D, Schrum AG (2012). IP-FCM measures physiologic protein-protein interactions modulated by signal transduction and small-molecule drug inhibition. PLoS One.

[32] Nicolini C, Fahnestock M (2018). The valproic acid-induced rodent model of autism. Exp Neurol.

[33] Lautz JD, Brown EA, AAW VS, SEP S. Synaptic activity induces input-specific rearrangements in a targeted synaptic protein interaction network. J Neurochem. 2018; 10.1111/jnc.14466. 10.1111/jnc.14466 PMC6150823 29804286

[34] Smith SE, Zhou YD, Zhang G, Jin Z, Stoppel DC, Anderson MP (2011). Increased gene dosage of Ube3a results in autism traits and decreased glutamate synaptic transmission in mice. Sci Transl Med.

[35] Rinaldi T, Kulangara K, Antoniello K, Markram H (2007). Elevated NMDA receptor levels and enhanced postsynaptic long-term potentiation induced by prenatal exposure to valproic acid. Proc Natl Acad Sci U S A.

[36] Wang CC, Held RG, Hall BJ (2013). SynGAP regulates protein synthesis and homeostatic synaptic plasticity in developing cortical networks. PLoS One.

[37] Ehninger D, Han S, Shilyansky C, Zhou Y, Li W, Kwiatkowski DJ, Ramesh V, Silva AJ (2008). Reversal of learning deficits in a Tsc2+/− mouse model of tuberous sclerosis. Nat Med.

[38] Kornau HC, Schenker LT, Kennedy MB, Seeburg PH (1995). Domain interaction between NMDA receptor subunits and the postsynaptic density protein PSD-95. Science.

[39] Irie M, Hata Y, Takeuchi M, Ichtchenko K, Toyoda A, Hirao K, Takai Y, Rosahl TW, Sudhof TC (1997). Binding of neuroligins to PSD-95. Science.

[40] Toft AK, Lundbye CJ, Banke TG (2016). Dysregulated NMDA-receptor signaling inhibits long-term depression in a mouse model of fragile X syndrome. J Neurosci.

[41] Guo W, Ceolin L, Collins KA, Perroy J, Huber KM (2015). Elevated CaMKIIalpha and hyperphosphorylation of Homer mediate circuit dysfunction in a fragile X syndrome mouse model. Cell Rep.

[42] Ronesi JA, Collins KA, Hays SA, Tsai NP, Guo W, Birnbaum SG, Hu JH, Worley PF, Gibson JR, Huber KM (2012). Disrupted Homer scaffolds mediate abnormal mGluR5 function in a mouse model of fragile X syndrome. Nat Neurosci.

[43] Gross C, Chang CW, Kelly SM, Bhattacharya A, McBride SM, Danielson SW, Jiang MQ, Chan CB, Ye K, Gibson JR (2015). Increased expression of the PI3K enhancer PIKE mediates deficits in synaptic plasticity and behavior in fragile X syndrome. Cell Rep.

[44] Gross C, Raj N, Molinaro G, Allen AG, Whyte AJ, Gibson JR, Huber KM, Gourley SL, Bassell GJ (2015). Selective role of the catalytic PI3K subunit p110beta in impaired higher order cognition in fragile X syndrome. Cell Rep.

[45] Hays SA, Huber KM, Gibson JR (2011). Altered neocortical rhythmic activity states in Fmr1 KO mice are due to enhanced mGluR5 signaling and involve changes in excitatory circuitry. J Neurosci.

[46] Poliak S, Salomon D, Elhanany H, Sabanay H, Kiernan B, Pevny L, Stewart CL, Xu X, Chiu SY, Shrager P (2003). Juxtaparanodal clustering of Shaker-like K+ channels in myelinated axons depends on Caspr2 and TAG-1. J Cell Biol.

[47] Anderson GR, Galfin T, Xu W, Aoto J, Malenka RC, Sudhof TC (2012). Candidate autism gene screen identifies critical role for cell-adhesion molecule CASPR2 in dendritic arborization and spine development. Proc Natl Acad Sci U S A.

[48] Krapivinsky G, Medina I, Krapivinsky L, Gapon S, Clapham DE (2004). SynGAP-MUPP1-CaMKII synaptic complexes regulate p38 MAP kinase activity and NMDA receptor-dependent synaptic AMPA receptor potentiation. Neuron.

[49] Sun J, Zhu G, Liu Y, Standley S, Ji A, Tunuguntla R, Wang Y, Claus C, Luo Y, Baudry M, Bi X (2015). UBE3A regulates synaptic plasticity and learning and memory by controlling SK2 channel endocytosis. Cell Rep.

[50] Roullet FI, Wollaston L, Decatanzaro D, Foster JA (2010). Behavioral and molecular changes in the mouse in response to prenatal exposure to the anti-epileptic drug valproic acid. Neuroscience.

[51] Zhang J, Liu LM, Ni JF (2017). Rapamycin modulated brain-derived neurotrophic factor and B-cell lymphoma 2 to mitigate autism spectrum disorder in rats. Neuropsychiatr Dis Treat.

[52] Nicolini C, Ahn Y, Michalski B, Rho JM, Fahnestock M (2015). Decreased mTOR signaling pathway in human idiopathic autism and in rats exposed to valproic acid. Acta Neuropathol Commun.

[53] Ethridge LE, White SP, Mosconi MW, Wang J, Pedapati EV, Erickson CA, Byerly MJ, Sweeney JA (2017). Neural synchronization deficits linked to cortical hyper-excitability and auditory hypersensitivity in fragile X syndrome. Mol Autism.

[54] Lovelace JW, Ethell IM, Binder DK, Razak KA (2018). Translation-relevant EEG phenotypes in a mouse model of fragile X syndrome. Neurobiol Dis.

[55] Rinaldi T, Silberberg G, Markram H (2008). Hyperconnectivity of local neocortical microcircuitry induced by prenatal exposure to valproic acid. Cereb Cortex.

[56] Rinaldi T, Perrodin C, Markram H (2008). Hyper-connectivity and hyper-plasticity in the medial prefrontal cortex in the valproic acid animal model of autism. Front Neural Circuits.

[57] Pawson T (2007). Dynamic control of signaling by modular adaptor proteins. Curr Opin Cell Biol.

[58] Ellegood J, Anagnostou E, Babineau BA, Crawley JN, Lin L, Genestine M, DiCicco-Bloom E, Lai JK, Foster JA, Penagarikano O (2015). Clustering autism: using neuroanatomical differences in 26 mouse models to gain insight into the heterogeneity. Mol Psychiatry.

[59] Darnell JC, Van Driesche SJ, Zhang C, Hung KY, Mele A, Fraser CE, Stone EF, Chen C, Fak JJ, Chi SW (2011). FMRP stalls ribosomal translocation on mRNAs linked to synaptic function and autism. Cell.

[60] Hadley D, Wu ZL, Kao C, Kini A, Mohamed-Hadley A, Thomas K, Vazquez L, Qiu H, Mentch F, Pellegrino R (2014). The impact of the metabotropic glutamate receptor and other gene family interaction networks on autism. Nat Commun.

[61] Kelleher RJ, Geigenmuller U, Hovhannisyan H, Trautman E, Pinard R, Rathmell B, Carpenter R, Margulies D (2012). High-throughput sequencing of mGluR signaling pathway genes reveals enrichment of rare variants in autism. PLoS One.

[62] Zhang J, Zhang JX, Zhang QL (2016). PI3K/AKT/mTOR-mediated autophagy in the development of autism spectrum disorder. Brain Res Bull.

[63] Sato A, Kasai S, Kobayashi T, Takamatsu Y, Hino O, Ikeda K, Mizuguchi M (2012). Rapamycin reverses impaired social interaction in mouse models of tuberous sclerosis complex. Nat Commun.

[64] Penagarikano O, Abrahams BS, Herman EI, Winden KD, Gdalyahu A, Dong H, Sonnenblick LI, Gruver R, Almajano J, Bragin A (2011). Absence of CNTNAP2 leads to epilepsy, neuronal migration abnormalities, and core autism-related deficits. Cell.

[65] Onda H, Lueck A, Marks PW, Warren HB, Kwiatkowski DJ (1999). Tsc2(+/−) mice develop tumors in multiple sites that express gelsolin and are influenced by genetic background. J Clin Invest.

[66] Young DM, Schenk AK, Yang SB, Jan YN, Jan LY (2010). Altered ultrasonic vocalizations in a tuberous sclerosis mouse model of autism. Proc Natl Acad Sci U S A.

[67] Potter WB, Basu T, O'Riordan KJ, Kirchner A, Rutecki P, Burger C, Roopra A (2013). Reduced juvenile long-term depression in tuberous sclerosis complex is mitigated in adults by compensatory recruitment of mGluR5 and Erk signaling. PLoS Biol.

[68] Consortium TD-BFX (1994). Fmr1 knockout mice: a model to study fragile X mental retardation. Cell.

[69] Kazdoba TM, Leach PT, Silverman JL, Crawley JN (2014). Modeling fragile X syndrome in the Fmr1 knockout mouse. Intractable Rare Dis Res.

[70] Giuffrida R, Musumeci S, D'Antoni S, Bonaccorso CM, Giuffrida-Stella AM, Oostra BA, Catania MV (2005). A reduced number of metabotropic glutamate subtype 5 receptors are associated with constitutive homer proteins in a mouse model of fragile X syndrome. J Neurosci.

[71] Martin HGS, Lassalle O, Brown JT, Manzoni OJ (2016). Age-dependent long-term potentiation deficits in the prefrontal cortex of the Fmr1 knockout mouse model of fragile X syndrome. Cereb Cortex.

[72] Rodier PM, Ingram JL, Tisdale B, Nelson S, Romano J (1996). Embryological origin for autism: developmental anomalies of the cranial nerve motor nuclei. J Comp Neurol.

[73] Schneider T, Przewlocki R (2005). Behavioral alterations in rats prenatally exposed to valproic acid: animal model of autism. Neuropsychopharmacology.

[74] Gandal MJ, Edgar JC, Ehrlichman RS, Mehta M, Roberts TP, Siegel SJ (2010). Validating gamma oscillations and delayed auditory responses as translational biomarkers of autism. Biol Psychiatry.

[75] Iossifov I, Ronemus M, Levy D, Wang Z, Hakker I, Rosenbaum J, Yamrom B, Lee YH, Narzisi G, Leotta A (2012). De novo gene disruptions in children on the autistic spectrum. Neuron.

[76] Rossi M, Chatron N, Labalme A, Ville D, Carneiro M, Edery P, des Portes V, Lemke JR, Sanlaville D, Lesca G (2017). Novel homozygous missense variant of GRIN1 in two sibs with intellectual disability and autistic features without epilepsy. Eur J Hum Genet.

[77] Barnby G, Abbott A, Sykes N, Morris A, Weeks DE, Mott R, Lamb J, Bailey AJ, Monaco AP, International molecular genetics study of autism C (2005). Candidate-gene screening and association analysis at the autism-susceptibility locus on chromosome 16p: evidence of association at GRIN2A and ABAT. Am J Hum Genet.

[78] Platzer K, Yuan H, Schutz H, Winschel A, Chen W, Hu C, Kusumoto H, Heyne HO, Helbig KL, Tang S (2017). GRIN2B encephalopathy: novel findings on phenotype, variant clustering, functional consequences and treatment aspects. J Med Genet.

[79] Iossifov I, Levy D, Allen J, Ye K, Ronemus M, Lee YH, Yamrom B, Wigler M (2015). Low load for disruptive mutations in autism genes and their biased transmission. Proc Natl Acad Sci U S A.

[80] Geisheker MR, Heymann G, Wang T, Coe BP, Turner TN, Stessman HAF, Hoekzema K, Kvarnung M, Shaw M, Friend K (2017). Hotspots of missense mutation identify neurodevelopmental disorder genes and functional domains. Nat Neurosci.

[81] Ramanathan S, Woodroffe A, Flodman PL, Mays LZ, Hanouni M, Modahl CB, Steinberg-Epstein R, Bocian ME, Spence MA, Smith M (2004). A case of autism with an interstitial deletion on 4q leading to hemizygosity for genes encoding for glutamine and glycine neurotransmitter receptor sub-units (AMPA 2, GLRA3, GLRB) and neuropeptide receptors NPY1R, NPY5R. BMC Med Genet.

[82] Jamain S, Quach H, Betancur C, Rastam M, Colineaux C, Gillberg IC, Soderstrom H, Giros B, Leboyer M, Gillberg C (2003). Mutations of the X-linked genes encoding neuroligins NLGN3 and NLGN4 are associated with autism. Nat Genet.

[83] Sanders SJ, He X, Willsey AJ, Ercan-Sencicek AG, Samocha KE, Cicek AE, Murtha MT, Bal VH, Bishop SL, Dong S (2015). Insights into autism spectrum disorder genomic architecture and biology from 71 risk loci. Neuron.

[84] Feyder M, Karlsson RM, Mathur P, Lyman M, Bock R, Momenan R, Munasinghe J, Scattoni ML, Ihne J, Camp M (2010). Association of mouse Dlg4 (PSD-95) gene deletion and human DLG4 gene variation with phenotypes relevant to autism spectrum disorders and Williams’ syndrome. Am J Psychiatry.

[85] Li J, Shi M, Ma Z, Zhao S, Euskirchen G, Ziskin J, Urban A, Hallmayer J, Snyder M (2014). Integrated systems analysis reveals a molecular network underlying autism spectrum disorders. Mol Syst Biol.

[86] Stessman HA, Xiong B, Coe BP, Wang T, Hoekzema K, Fenckova M, Kvarnung M, Gerdts J, Trinh S, Cosemans N (2017). Targeted sequencing identifies 91 neurodevelopmental-disorder risk genes with autism and developmental-disability biases. Nat Genet.

[87] Durand CM, Betancur C, Boeckers TM, Bockmann J, Chaste P, Fauchereau F, Nygren G, Rastam M, Gillberg IC, Anckarsater H (2007). Mutations in the gene encoding the synaptic scaffolding protein SHANK3 are associated with autism spectrum disorders. Nat Genet.

[88] Nurmi EL, Bradford Y, Chen Y, Hall J, Arnone B, Gardiner MB, Hutcheson HB, Gilbert JR, Pericak-Vance MA, Copeland-Yates SA (2001). Linkage disequilibrium at the Angelman syndrome gene UBE3A in autism families. Genomics.

[89] Glessner JT, Wang K, Cai G, Korvatska O, Kim CE, Wood S, Zhang H, Estes A, Brune CW, Bradfield JP (2009). Autism genome-wide copy number variation reveals ubiquitin and neuronal genes. Nature.

[90] Hamdan FF, Daoud H, Piton A, Gauthier J, Dobrzeniecka S, Krebs MO, Joober R, Lacaille JC, Nadeau A, Milunsky JM (2011). De novo SYNGAP1 mutations in nonsyndromic intellectual disability and autism. Biol Psychiatry.

[91] Riviere JB, Mirzaa GM, O'Roak BJ, Beddaoui M, Alcantara D, Conway RL, St-Onge J, Schwartzentruber JA, Gripp KW, Nikkel SM (2012). De novo germline and postzygotic mutations in AKT3, PIK3R2 and PIK3CA cause a spectrum of related megalencephaly syndromes. Nat Genet.

[92] Serajee FJ, Nabi R, Zhong H, Mahbubul Huq AH (2003). Association of INPP1, PIK3CG, and TSC2 gene variants with autistic disorder: implications for phosphatidylinositol signalling in autism. J Med Genet.

