# Supplementary material for: Clustering the autisms using glutamate synapse protein interaction networks from cortical and hippocampal tissue of seven mouse models
Source: Mol Autism. 2018 Sep 15;9:48. doi: 10.1186/s13229-018-0229-1 (PMC6139139; doi:10.1186/s13229-018-0229-1)
Supplement: Supplementary file 2 — Figure S1. AKT phosphorylation is reduced in VPA mice but normal in all other models examined. Related to Fig. 6 (A) Representative western blots of cortical samples from adult mice probed with the indicated antibodies. (B) Quantification, axes match Fig. 6. N = 3–10 individuals per genotype. *p < 0.001 by two-tailed t test, Bonferroni-corrected for multiple comparisons. (PDF 194 kb) [file 13229_2018_229_MOESM2_ESM.pdf]

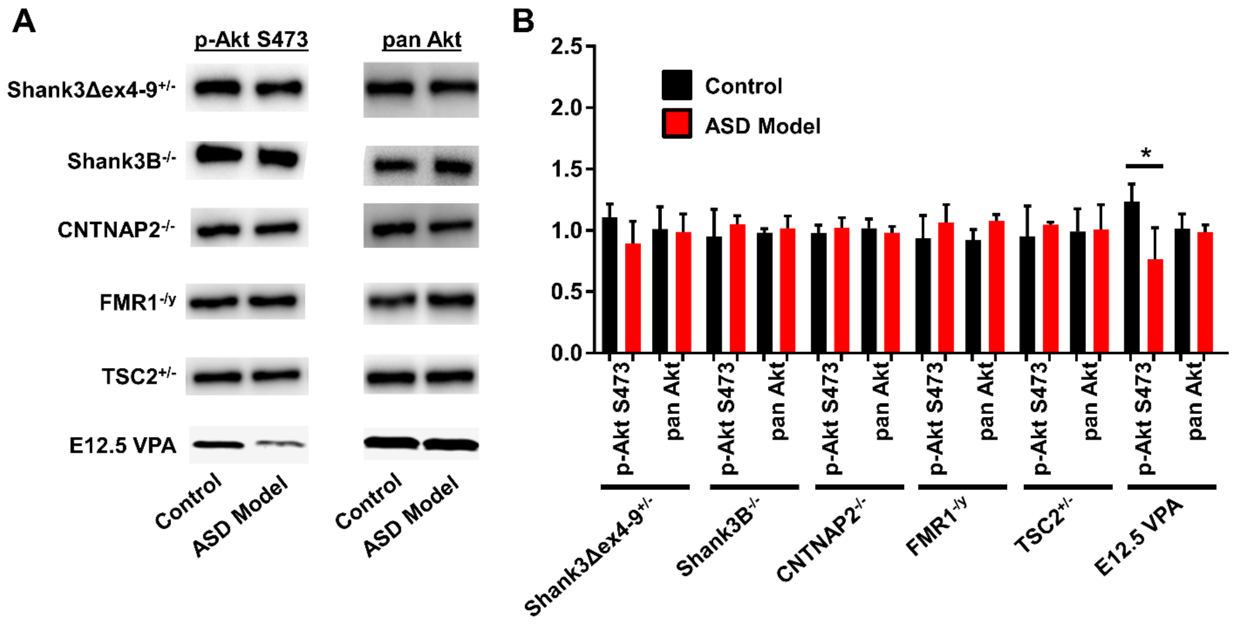

Figure S1: AKT phosphorylation is reduced in VPA mice but normal in all other models examined. Related to Figure 6 A) Representative western blots of cortical samples from adult mice probed with the indicated antibodies. B) Quantification, axes match Figure 6. N = 3-10 individuals per genotype. * $p < 0.001$ by 2-tailed t test, Bonferroni-corrected for multiple comparisons.
